# Datasets describing the growth and molecular features of hepatocellular carcinoma patient-derived xenograft cells grown in a three-dimensional macroporous hydrogel

**DOI:** 10.1016/j.dib.2018.03.045

**Published:** 2018-03-17

**Authors:** Eliza Li Shan Fong, Tan Boon Toh, Quy Xiao Xuan Lin, Zheng Liu, Lissa Hooi, Masturah Bte Mohd Abdul Rashid, Touati Benoukraf, Edward Kai-Hua Chow, The Hung Huynh, Hanry Yu

**Affiliations:** a Department of Biomedical Engineering, National University of Singapore, Singapore; b Cancer Science Institute of Singapore, National University of Singapore, Singapore; c Institute of Bioengineering and Nanotechnology, Agency for Science, Technology and Research (A^⁎^STAR) Singapore; d National Cancer Center Singapore, Singapore; e Department of Physiology, Yong Loo Lin School of Medicine, National University of Singapore, Singapore; f Mechanobiology Institute, National University of Singapore, Singapore; g BioSyM, Singapore-MIT Alliance for Research and Technology, Singapore; h Department of Gastroenterology, Nanfang Hospital, Southern Medical University, Guangzhou, China; i NUS Graduate School of Integrative Sciences and Engineering, National University of Singapore, Singapore; j Department of Pharmacology, Yong Loo Lin School of Medicine, National University of Singapore, Singapore

## Abstract

This data article presents datasets associated with the research article entitled “Generation of matched patient-derived xenograft *in vitro–in vivo* models using 3D macroporous hydrogels for the study of liver cancer” (Fong et al., 2018) [1]. A three-dimensional macroporous sponge system was used to generate *in vitro* counterparts to various hepatocellular carcinoma patient-derived xenograft (HCC-PDX) lines. This article describes the viability, proliferative capacity and molecular features (genomic and transcriptomic profiles) of the cultured HCC-PDX cells. The sequencing datasets are made publicly available to enable critical or further analyzes.

**Specifications Table**

**Table:** 

Subject area	Biology
More specific subject area	Development of HCC-PDX *in vitro* models
Type of data	Graphs, Tables, Sequencing data
How data was acquired	Microscope: Olympus Fluoview FV1000 or Zeiss LSM 710 confocal microscope
	XPS: VG ESCALAB Mk II spectrometer
	Whole exome sequencing: HiSeq. 4000 (150PE); RNA-sequencing: HiSeq. 2500 1T(100PE)
Data format	Analyzed and raw
Experimental factors	Cells from various HCC-PDX *in vivo* models were cultured in a 3D sponge scaffold to determine whether these cells could be grown *in vitro.*
Experimental features	The degree of genomic and transcriptomic correlation between paired *in vitro* and *in vivo* HCC-PDX models was established for all the lines.
Data source location	Singapore
Data accessibility	GEO database (GSE109955)

**Value of the data**

• The data presents the molecular features (genomic and transcriptomic profiles) of various *in vivo* and corresponding *in vitro* HCC-PDX models and could be used by other researchers to study HCC.
• This data allows for other researchers to extend the correlative analyses between the established *in vivo* and corresponding *in vitro* HCC-PDX models.
• The whole exome sequencing (WES) and RNA-sequencing (RNA-seq) data may also allow for comparisons between the HCC-PDX models and other HCC models to be made.

## 1 Data

**Fig. 1:**
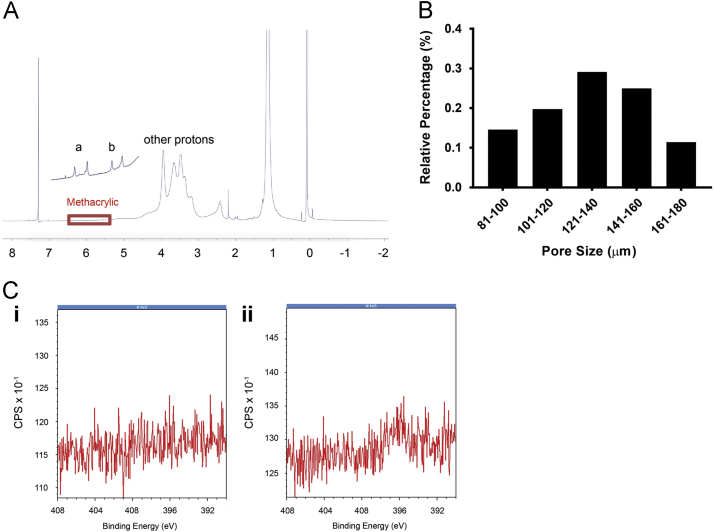
**(A)** NMR spectrum of MA-HPC. **(B)** Pore size distribution of MA-HPC sponge. **(C) i.** X-ray photoelectron spectrum of MA-HPC sponge without galactosamine conjugation. **ii.** X-ray photoelectron spectrum of MA-HPC sponge after galactosamine conjugation. Increased nitrogen atomic counts is observed at 396 eV.

**Fig. 2:**
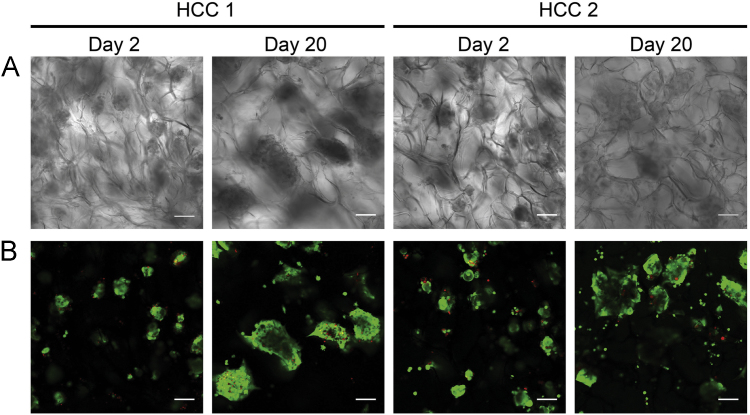
**(A)** Brightfield and **(B)** fluorescence images of HCC1-3DPDX and HCC2-3DPDX at Day 2 and 20 in culture. Viable cells are stained green by calcein-AM and dead cells are stained red by propidium-iodide. Scale bars = 100 µm.

**Fig. 3:**
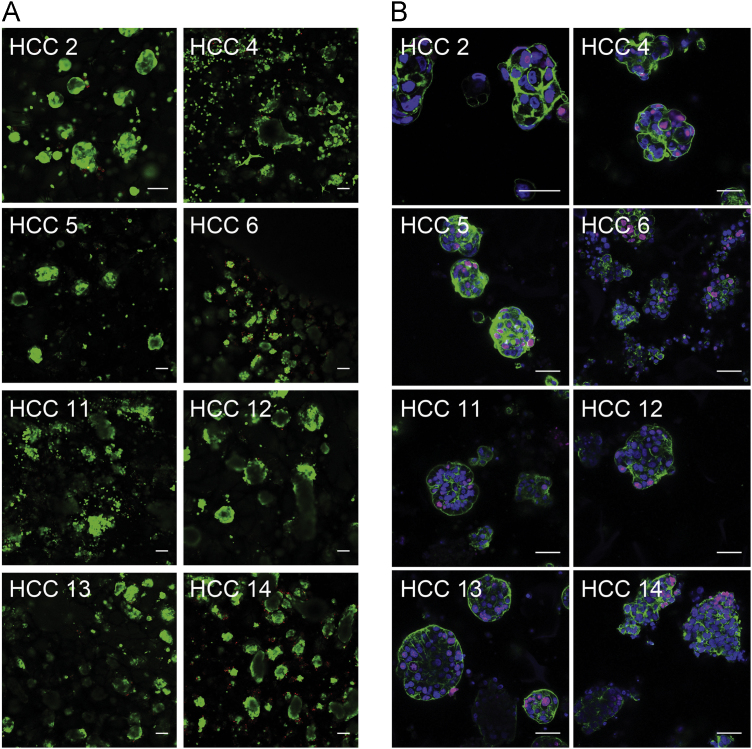
**(A)** Fluorescence images of 8 HCC-3DPDX lines assessed for viability in culture. Cells were stained with calcein-AM and propidium iodide at Day 7. Scale bars = 100 µm. **(B)** Ki-67 expression in the same cultures at Day 7. Ki-67^+^ cells are in magenta; cells were also stained with DAPI (blue) and phalloidin (green) to visualize the nucleus and actin cytoskeleton, respectively. Scale bars = 50 µm.

**Fig. 4:**
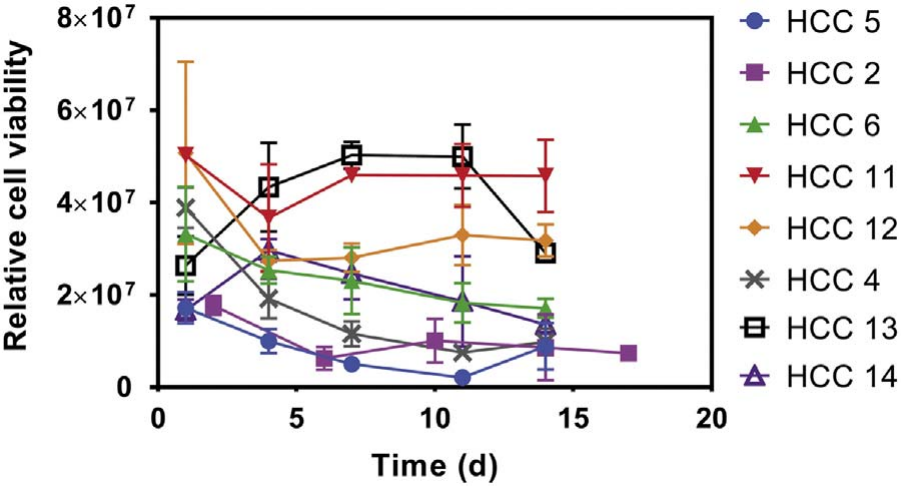
Relative viability of cells derived from other 8 HCC-3DPDX lines cultured in MA-HPC sponge over two weeks in culture, as indicated by ATP content using CellTiter-Glo®.

The dataset of this article provides information on the characteristics of the HCC-PDX cells when grown in the sponge system. Fig. 1 describes the physical characterization of the sponge system used to culture the HCC-PDX cells. Fig. 2, Fig. 3, Fig. 4 describes the viability, proliferative capacity and growth profile of 8 different HCC-PDX lines grown in the sponge (data for 6 other lines can be found in [1]). Following these data are those comparing the SNP and INDEL overlap (Table 2) and mutational signature of HCC-PDX cells grown *in vitro* versus those *in vivo* (Fig. 6), as well as the correlation analysis of gene expression levels (based on a known set of dysregulated HCC genes as reported by Ho et al. [2]) between individual HCC-PDX *in vivo-in vitro* pairs (Fig. 8).

## 2 Experimental design, materials and methods

For each HCC-PDX line, HCC-PDX cells were harvested from the tumors, dissociated and seeded onto macroporous sponge. After 7 days in culture, RNA and DNA were extracted for WES and RNA-seq. HCC-PDX cells grown in the sponge will be referred to as HCC3D-PDX, while their corresponding *in vivo* counterparts will be referred to as HCC-PDX.

### 2.1 Material characterization of macroporous sponge

Hydroxypropylcellulose (HPC) was used to fabricate the macroporous sponge. In brief, HPC is first grafted with methacrylic (MA) groups, which renders the polymer photo-crosslinkable. NMR spectroscopy was performed to determine the successful grafting of methacrylic (MA) groups onto HPC (Fig. 1**A**). Subsequently, MA-HPC is allowed to undergo thermal-induced phase separation and cross-linked with gamma irradiation. Following which, pore size distribution of the resulting sponge was quantified with ImageJ software from collective top view images of sponge obtained using scanning electron microscopy (Fig. 1**B**). Top views of the sponge surface morphology were captured using SEM (JEOL JSM-5600, Japan) at 5 kV. Prior to imaging, the dried sponge was sputter-coated with platinum for 60 s. Sponge was also conjugated with galactose moieties as previously described [3]. The successful conjugation of galactose onto the MA-HPC backbone was confirmed by X-ray photoelectron spectroscopy (Fig. 1**C**). Measurements were made on a VG ESCALAB Mk II spectrometer with a MgKa X-ray source (1253.6 eV photons) at a constant retard ratio of 40.

### 2.2 Viability, growth and proliferative capacity of HCC-PDX cells in sponge

The ability of 14 different HCC-PDX lines to grow in this macroporous sponge is reported in [1]. Fig. 2 shows the morphology and viability of 2 different HCC-PDX lines grown in the sponge at Day 2 and 20. Cells were stained with calcein-AM and propidium iodide which labels live cells green, and dead cells red. Samples were assessed for viability using calcein-AM (2 µM) and propidium iodide (25 µg/mL). Following a 30 min incubation with calcein-AM and propidium iodide, samples were immediately imaged using a Olympus Fluoview FV1000 or Zeiss LSM 710 confocal microscope. Samples were assessed for growth using CellTiter-Glo (Promega) as described by the manufacturer. While the viability, proliferative capacity and growth profile of 6 HCC-PDX lines were reported in [1], this article illustrates that of the other 8 HCC-PDX lines grown in the sponge (Fig. 3, Fig. 4).

### 2.3 Transcriptomic correlation between HCC-PDX and HCC3D-PDX using RNA-seq

#### 2.3.1 RNA-seq library preparation

RNA quality was assessed by analysis of rRNA band integrity on an Agilent RNA 6000 Nano kit (Agilent Technologies, CA). Before cDNA library construction, 1 µg of total RNA and magnetic beads with Oligo (dT) were used to enrich for poly(A) mRNA. Then, the purified mRNAs were disrupted into short fragments, and the double-stranded cDNAs were immediately synthesized. The cDNAs were subjected to end repair, poly(A) addition, and connection with sequencing adapters using the TruSeq RNA sample prep Kit (Illumina, CA). The suitable fragments automatically purified by BluePippin 2% agarose gel cassette (Sage Science, MA) were selected as templates for PCR amplification. The final library sizes and qualities were evaluated electrophoretically with an Agilent High Sensitivity DNA kit (Agilent Technologies, CA) and the fragment was found to be between 350 and 450 bp. Subsequently, the library was sequenced using an Illumina HiSeq. 2500 sequencer (Illumina, CA, Table 1).

**Table 1:** Sequencing read numbers in RNA-seq and WES.

Sample ID	PDX		3DPDX	
	RNA-seq	WES	RNA-seq	WES
HCC1	33,265,889	48,058,115	31,205,689	51,644,536
HCC2	35,736,459	60,744,719	33,340,192	50,997,100
HCC3	33,474,544	48,698,816	33,163,355	50,428,623
HCC4	35,046,413	46,771,662	35,242,246	50,703,953
HCC5	33,880,630	54,662,902	36,255,320	52,446,010
HCC6	33,769,914	57,642,613	34,367,687	51,857,840
HCC7	45,698,704	53,945,098	31,841,998	51,155,417
HCC8	32,886,967	55,446,768	35,257,904	51,633,096
HCC9	43,028,572	NA	34,488,849	NA
HCC10	39,235,661	NA	33,741,181	NA
HCC11	40,107,949	56,874,910	30,719,613	50,529,040
HCC12	35,585,487	NA	30,727,805	NA
HCC13	32,583,039	45,400,907	35,238,020	52,108,340
HCC14	32,798,859	61,435,067	32,526,214	51,987,242

#### 2.3.2 RNA-seq processing

**Fig. 5:**
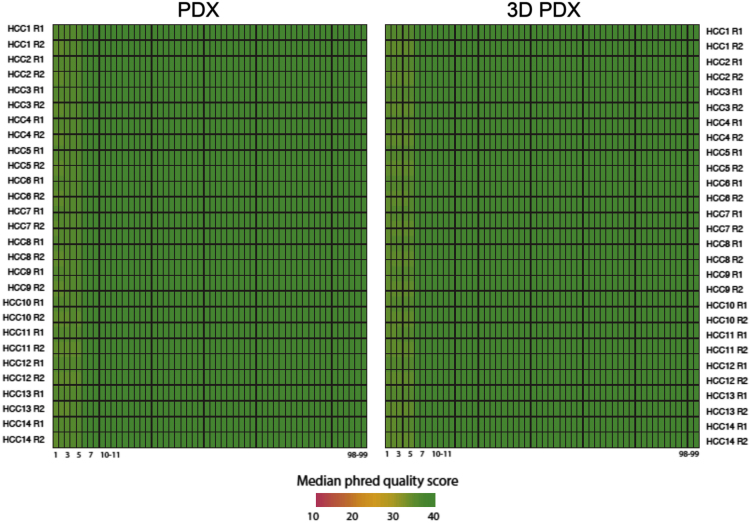
Sequencing quality check for RNA-sequencing datasets. Heatmaps show the median phred quality scores of reads RNA-seq experiments for PDX (left) and 3DPDX (right). Each row represents an end of each paired-end read (R1 and R2), while each column represents the sequenced nucleotide position in the read.

After quality check with FastQC (Fig. 5), short reads were aligned to human genome assembly hg38 using STAR [4]. Transcript expression levels were measured as Fragments Per Kilobase of transcript per Million mapped reads (FPKM) using the analyzeRepeats.pl script from the HOMER package [5].

#### 2.3.3 Transcriptomic correlation between the matched PDX and 3DPDX models

To investigate whether HCC-PDX and HCC-3DPDX share similar gene expression profiles, we focused on 219 up-regulated (EPR1 reported by Ho et al. has been discontinued since 2011 [6]) and 514 down-regulated genes known to be dysregulated in HCC [2]. Comparative analysis was performed using Pearson correlation (gene expression measure FPKM underwent inverse hyperbolic sine transformation). Fig. 8 shows the degree of correlation between paired *in vivo* and *in vitro* models for the 14 HCC-PDX lines. RNA-seq data for the 14 HCC-PDX lines (both *in vivo* and corresponding *in vitro* models) is publicly available in the GEO datasets (GSE109903).

### 2.4 Genomic profiling of HCC-PDX and HCC3D-PDX using WES

#### 2.4.1 WES library preparation

The quality and quantity of purified DNA were assessed by fluorometry (Qubit, Invitrogen) and gel electrophoresis. Briefly, 500ng of genomic DNA from each sample was fragmented by acoustic shearing on a Covaris S2 instrument. Fragments in 150–300 bp were ligated to Illumina's adapters and PCR-amplified. The samples were concentrated to 300 ng in 3.4 μl DW using a Speedvac machine (Thermo Scientific) and hybridized with RNA probes, SureSelect XT Human All Exon V5 Capture library for 16–24 h at 65 °C. After hybridization, the captured targets were pulled down by biotinylated probe/target hybrids using streptavidin-coated magnetic beads (Dynabeads My One Streptavidine T1; Life Technologies Ltd.) and buffers. The selected regions were then PCR-amplified using Illumina PCR primers. Libraries were quantified using the Agilent 2100 Bioanalyzer (Agilent Technologies) and KAPA Library Quantification Kit (KK4824, Kapa Biosystems). The resulting purified libraries were applied to an Illumina flow cell for cluster generation and sequenced using 150 bp paired-end reads on an Illumina Hiseq. 2500 sequencer by following the manufacturer's protocols (Table 1). Image analysis were performed using the HiSeq control Software version 1.8.4.

#### 2.4.2 WES processing

In order to remove mouse reads in PDX samples, BBMap [7] was applied to the fastq files based on hg19 and Ensembl Release 77 reference genome for human and mouse, respectively, and the reads classified into human reads only were then analyzed. After quality check by FastQC (Fig. 7), reads in high quality were aligned to human reference genome hg19 using Burrows Wheeler Aligner (BWA) [8] and duplicated reads were removed using Picard. Improvement of alignments and genetic variants calling were completed using Genome Analysis Toolkit (GATK) [9].

#### 2.4.3 Genomic profiling of the matched PDX and 3DPDX models

**Fig. 6:**
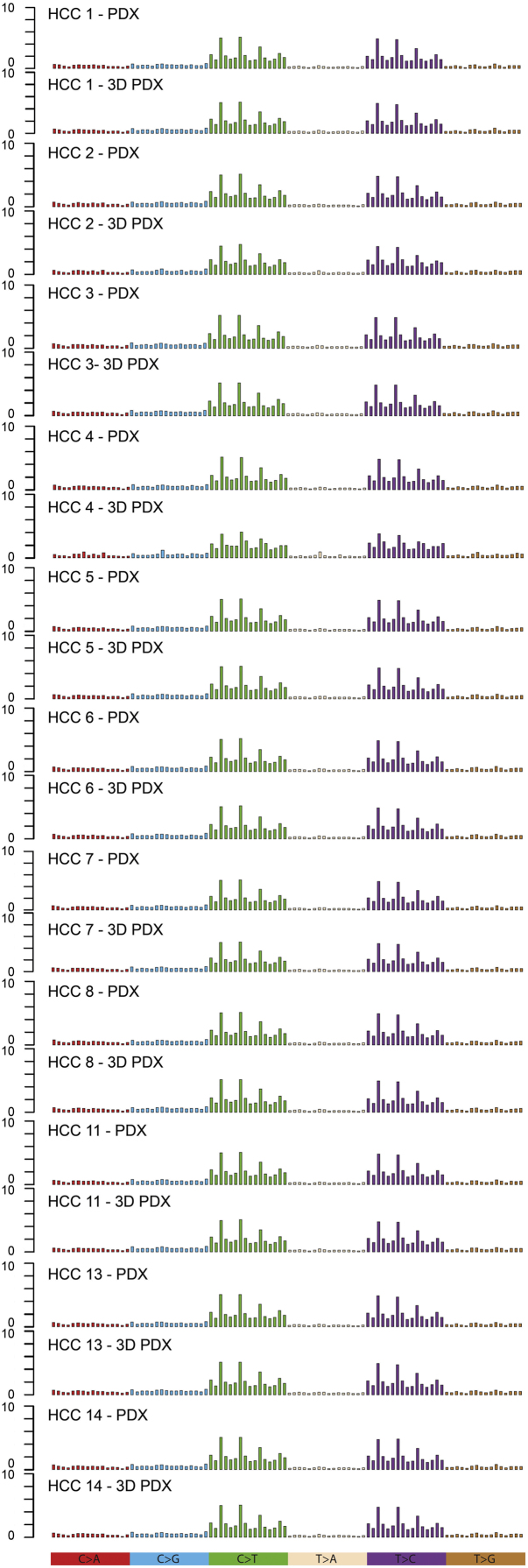
Mutational signature of *in vitro* and *in vivo* HCC-PDX. Six main types of substitutions, namely C > A, C > G, C > T, T > A, T > C and T > G were extracted.

Ovelapping of SNP and INDEL between HCC-PDX and HCC-3DPDX were analyzed using VCFtools [10]. Common SNP and INDEL overlap between HCC-PDX and HCC3D-PDX are shown in Table 2. In order to profile substitution patterns for signature [11] in HCC-PDX and HCC-3DPDX, we extracted 6 main types of substitutions, namely C > A, C > G, C > T, T > A, T > C and T > G. Specifically, for each main nucleotide substitution type, there are 16 different trinucleotide combinations and the occurrence frequency of each trinucleotide-based substitution subtype was calculated. Mutational signature for paired *in vivo-in vitro* HCC-PDX models is shown in Fig. 6. Whole exome sequencing data for 11 HCC-PDX lines (both *in vivo* and corresponding *in vitro* models) is publicly available in the GEO datasets (GSE109954).

**Fig. 7:**
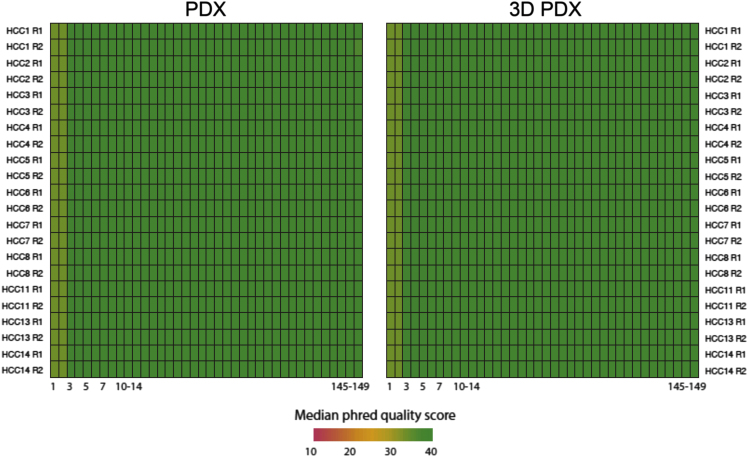
Sequencing quality check for WES datasets. Heatmaps show the median phred quality scores of reads in WES experiments for PDX (left) and 3DPDX (right). Each row represents an end of each paired-end read (R1 and R2), while each column represents the sequenced nucleotide position in the read.

**Fig. 8:**
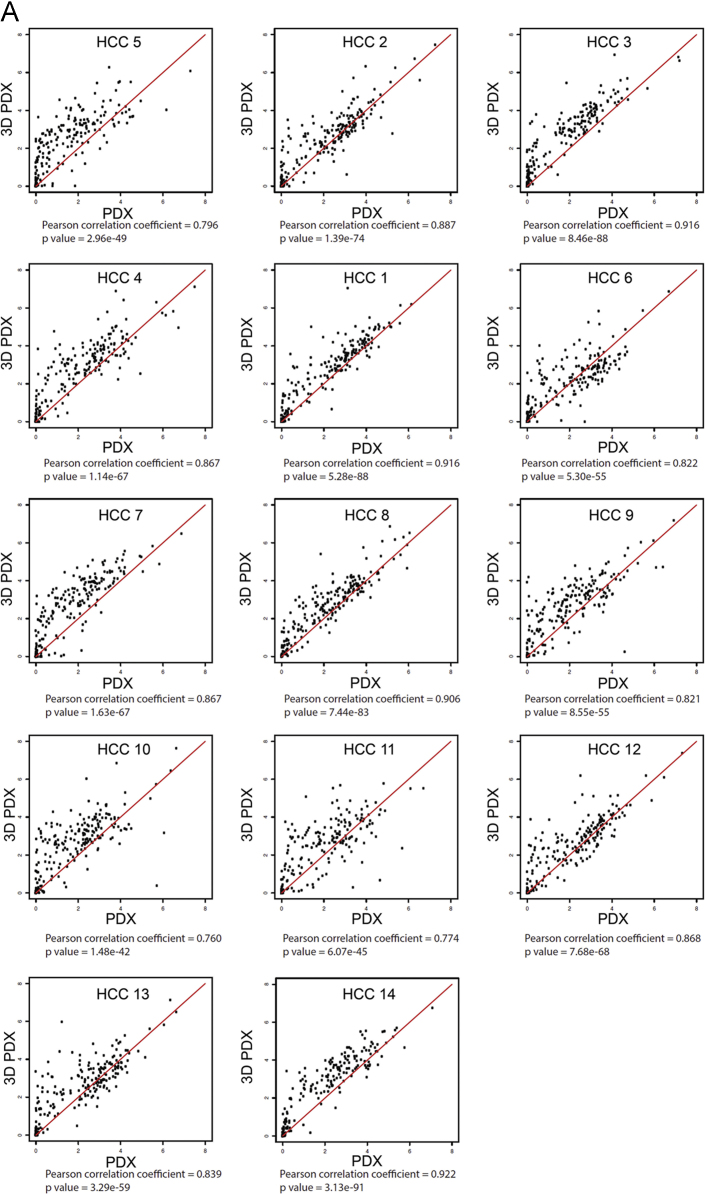

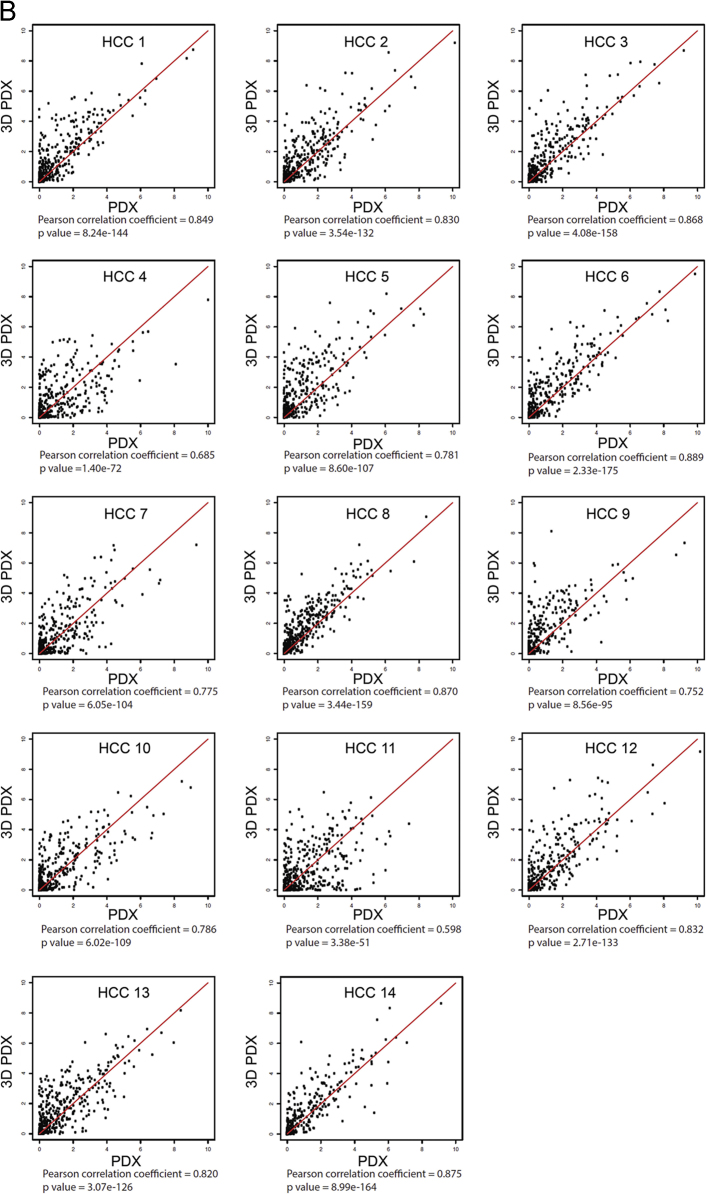
Correlation analysis details between HCC-PDX and HCC-3DPDX for known **(A)** up-regulated and **(B)** down-regulated genes in HCC.

**Table 2:** Common SNP and Indel overlap between PDX and 3D PDX in 11 HCC lines.

Sample ID	Type	SNP			Indel		
		Detected SNP number	Common SNP number	Common SNP percentage (%)	Detected indel number	Common indel number	Common indel percentage (%)
HCC1	PDX	40,914	40,352	98.63	3075	2927	95.19
	PDX3D	41,225		97.88	3126		93.63
							
HCC2	PDX	42,523	41,790	98.28	3219	3018	93.76
	PDX3D	51,701		80.83	3215		93.87
							
HCC3	PDX	42,810	42,153	98.47	3322	3158	95.06
	PDX3D	43,165		97.66	3361		93.96
							
HCC4	PDX	41,758	40,844	97.81	3323	3106	93.47
	PDX3D	85,664		47.68	3296		94.24
							
HCC5	PDX	42,267	41,557	98.32	3277	3082	94.05
	PDX3D	42,148		98.60	3266		94.37
							
HCC6	PDX	42,944	42,221	98.32	3306	3037	91.86
	PDX3D	42,831		98.58	3218		94.38
							
HCC7	PDX	42,174	41,414	98.19	3313	3094	93.39
	PDX3D	43,277		95.70	3204		96.57
							
HCC8	PDX	42,135	41,348	98.13	3105	2889	93.04
	PDX3D	42,065		98.30	3086		93.62
							
HCC11	PDX	41,742	40,920	98.03	3301	3051	92.43
	PDX3D	42,663		95.91	3164		96.43
							
HCC13	PDX	41,416	40,829	98.58	3178	3030	95.34
	PDX3D	41,561		98.24	3232		93.75
							
HCC14	PDX	41,236	40,469	98.14	3254	3063	94.13
	PDX3D	41,868		96.66	3182		96.26
